# Dietary behaviors related to cancer prevention among pre-adolescents and adolescents: the gap between recommendations and reality

**DOI:** 10.1186/1475-2891-10-60

**Published:** 2011-06-01

**Authors:** Dawn M Holman, Mary C White

**Affiliations:** 1 Epidemiology and Applied Research Branch, Division of Cancer Prevention and Control, Centers for Disease Control and Prevention, 4770 Buford Highway NE, Mailstop K-55, Atlanta, GA 30341, USA

## Abstract

**Background:**

Diet is thought to play an important role in cancer risk. This paper summarizes dietary recommendations for cancer prevention and compares these recommendations to the dietary behaviors of U.S. youth ages 8-18.

**Methods:**

We identified cancer prevention-related dietary recommendations from key health organizations and assessed dietary consumption patterns among youth using published statistics from the National Health and Nutrition Examination Survey, the national Youth Risk Behavior Survey, and other supplemental sources.

**Results:**

Cancer prevention guidelines recommend a diet rich in fruits, vegetables, and whole grains, recommend limiting sugary foods and beverages, red and processed meats, sodium, and alcohol, and recommend avoiding foods contaminated with carcinogens. However, youth typically do not meet the daily recommendations for fruit, vegetable, or whole grain consumption and are over-consuming energy-dense, sugary and salty foods.

**Conclusions:**

A large discrepancy exists between expert recommendations about diet and cancer and actual dietary practices among young people and points to the need for more research to better promote the translation of science into practice. Future research should focus on developing and evaluating policies and interventions at the community, state and national levels for aligning the diets of youth with the evolving scientific evidence regarding cancer prevention.

## Introduction

The consensus among scientists in the field of cancer control is that diet plays an important but not fully understood role in modifying cancer risk and that certain changes in the American diet could have a beneficial impact on cancer occurrence [1-6]. However, diets are complex, and different components in the diet have been hypothesized to operate through different mechanisms throughout the process of cancer development. Accurately measuring dietary intake also poses many methodological challenges [7]. As a result, the contribution of diet to cancer risk is difficult to estimate with any degree of scientific certainty [8-10]. Although the benefits may be difficult to quantify, it has been proposed that efforts to align dietary consumption patterns among American youth with cancer prevention recommendations could reduce lifetime cancer risk as well as improve overall health [4,11]. This paper summarizes current dietary recommendations for cancer prevention and compares these recommendations to the dietary behaviors of pre-adolescents and adolescents in the United States (U.S.). Identifying gaps between dietary recommendations and the diets of youth will help to inform and direct future research and intervention efforts.

## Methods

### Data sources and study selection

We used a targeted approach to identify the most recent cancer prevention-related dietary recommendations of key organizations, including the American Academy of Pediatrics (AAP), American Cancer Society (ACS), American Heart Association (AHA), American Institute for Cancer Research (AICR), International Agency for Research on Cancer (IARC), U.S. Department of Health and Human Services (HHS), U.S. Department of Agriculture (USDA), World Cancer Research Fund International (WCRF), and World Health Organization (WHO). We used National Institutes of Health (NIH) PubMed and the National Guideline Clearinghouse to search for additional information on dietary recommendations published from 2000 to present.

To assess dietary patterns among youth in the U.S., we used published statistics based on data from the National Health and Nutrition Examination Survey (NHANES) [12] and the national Youth Risk Behavior Survey (YRBS) [13], as well as supplementary information available on the USDA website [14]. NHANES is a continuous, nationally representative, cross-sectional survey. A dietary interview component of NHANES is conducted as a partnership between USDA and HHS [15]. Two in-person, 24-hour food recall interviews are conducted with each individual [15]. Additional information on the dietary collection methods is available elsewhere [12,15]. NHANES body mass index (BMI) data comes from height and weight measurements taken during data collection [15]. The sample sizes for the various NHANES statistics cited in this paper ranged from 1667 to 6087 youth within the age range of 12-19 years [16-23]. The national YRBS is a school-based survey that has been conducted since 1990 and uses a three-stage cluster sample design to produce a nationally representative sample of students in grades 9-12 [13]. Students complete the self-administered questionnaire in school during one class period. The national YRBS includes questions about fruit, vegetable, and soda consumption in the past seven days, lifetime alcohol use, alcohol use in the past thirty days, height, weight, and self-described weight status, in addition to questions about other health-risk behaviors [13]. The statistics cited in this paper from the 2009 national YRBS included 16,410 students from 158 schools [13]. Published results include current prevalence rates of health-risk behaviors and information on changes over time for each variable based on logistic regression analyses that controlled for sex, grade, and race/ethnicity and that simultaneously assessed linear and quadratic time effects [13]. We obtained information regarding youth exposure to carcinogenic food contaminants from the *Fourth National Report on Human Exposure to Environmental Chemicals*, a CDC publication which uses blood and urine samples collected as part of NHANES to assess exposure of the U.S. population to chemicals in the environment [24].

### Data extraction

We grouped dietary recommendations for cancer prevention by dietary factors. For example, we grouped all recommendations regarding fruit consumption together and all recommendations regarding consumption of red and processed meat together. We further organized recommendations by association with a decreased cancer risk and association with an increased cancer risk. We extracted corresponding statistics on dietary behaviors of youth from the previously described sources.

## Results

Current dietary guidelines and recommendations include recommendations that are thought to be relevant for reducing lifetime cancer risk. These guidelines and recommendations are based primarily on expert opinion, informed largely by observational epidemiologic studies that have linked dietary factors with either an increased or decreased risk in one or more cancers in adults. For cancer prevention, the focus has been on increasing the consumption of foods associated with a decreased cancer risk and limiting or avoiding foods associated with an increased cancer risk.

Foods thought to be associated with a decreased cancer risk include fruits, vegetables, and unprocessed, whole grains, and current recommendations for these foods are summarized in Table 1[1-3,25,26]. In addition, guidelines to reduce the development of obesity in children are indirectly related to cancer prevention, because obesity has been associated with cancer occurrence at several sites among adults [1-3,27-29]. Dietary guidelines to prevent obesity emphasize a reduction in the consumption of foods with low nutritional value and high energy density, such as refined sugars and sugar-sweetened beverages [25,26].

**Table 1:** Current recommendations for dietary factors associated with reduced cancer risk

Factor	Recommendation	Source
**Fruit and vegetable consumption**		
• Eat five or more servings of a variety of vegetables and fruits each day.		ACS [3]
• Eat vegetables and fruits daily; limit juice intake.		AHA [25]
• Eat at least five servings (at least 400 g or 14 oz) of a variety of non-starchy vegetables and of fruits every day.		AICR [1]
• Consume sufficient amounts of fruits and vegetables while staying within energy needs; choose a variety of fruits and vegetables each day.		DGA [26]
• Have a diet which includes at least 400 g per day of total fruits and vegetables.		WHO [6]
**Whole grain consumption**		
• Chose whole grains in preference to processed (refined) grains and sugars.		ACS [3]
• Eat whole grain breads and cereals rather than refined grain products.		AHA [25]
• Eat relatively unprocessed grains and/or legumes with every meal.		AICR [1]
• Consume 3 or more ounce-equivalents of whole-grain products per day; At least half of grains consumed should come from whole grains.		DGA [26]
**Healthy weight**		
• Choose foods and beverages in amounts that help achieve and maintain a healthy weight through life.		ACS [3]
• Balance dietary calories with physical activity to maintain normal growth.		AHA [25]
• Be as lean as possible within the normal range of body weight (BMI of 18.5-24.9 kg/m^2^).		AICR [1]
• To maintain body weight in a healthy range, balance calories from foods and beverages with calories expended.		DGA [26]
• Maintain weight such that BMI is in the range of 18.5-24.9 kg/m^2^, and avoid weight gain during adult life.		WHO [6]

The consumption of some food items may increase the risk of cancer because of the presence of carcinogenic substances in these items, either naturally occurring or resulting from storage or preparation. Foods thought to be associated with an increased cancer risk include red and processed meats, salt, alcohol, foods contaminated with mycotoxins such as aflatoxin, and arsenic-contaminated water [1-3,25,26]. Current recommendations for food items associated with an increased cancer risk are summarized in Table 2.

**Table 2:** Current recommendations for dietary factors associated with increased cancer risk

Factor	Recommendation	Source
**Red and processed meat**		
• Limit consumption of processed and red meats.		ACS [3]
• People who eat red meat to consume less than 500 g (18 oz) a week, very little if any to be processed.		AICR [1]
• When selecting and preparing meat, make choices that are lean, low-fat, or fat-free.		DGA [26]
• Those who are not vegetarian are advised to moderate consumption of preserved meat.		WHO [6]
**Refined sugars**		
• Limit consumption of refined carbohydrates.		ACS [3]
• Reduce the intake of sugar-sweetened beverages and foods.		AHA [25]
• Avoid sugary drinks. Limit refined, starchy foods.		AICR [1]
• Choose and prepare foods and beverages with little added sugars or caloric sweeteners.		DGA [26]
**Energy-dense foods**		
• Limit consumption of energy-dense foods. Consume 'fast foods' sparingly, if at all.		AICR [1]
**Salt**		
• Avoid salt-preserved, salted, or salty foods; preserve foods without using salt. Limit consumption of processed foods with added salt to ensure an intake of less than 6 g (2.4 g sodium) a day.		AICR [1]
• Reduce salt intake, including salt from processed foods.		AHA [25]
• Consume less than 2,300 mg (approximately 1 teaspoon of salt) of sodium per day.		DGA [26]
• Overall consumption of salt-preserved foods and salt should be moderate.		WHO [6]
**Alcohol**		
• Drink no more than one drink per day for women or two per day for men.		ACS [3]
• Limit alcohol consumption to no more than two drinks for men and one for women a day.		AICR [1]
• Alcohol should not be consumed by children and adolescents.		DGA [26]
• Consumption of alcoholic beverages is not recommended: if consumed, do not exceed two units^g ^per day.		WHO [6]
**Mycotoxins**		
• Do not eat moldy grains or legumes.		AICR [1]
• Minimize exposure to aflatoxin in foods.		WHO [6]
**Arsenic**		
• Avoid use of any source of water that may be contaminated with arsenic.		AICR [1]

### Fruits and vegetables

Although current guidelines emphasize a diet rich in fruits and vegetables [1-3,5,6,25,26], such recommendations are not reflected in the diets of American youth [13,16,17]. The 2005 *Dietary Guidelines for Americans *[DGA] provide fruit and vegetable recommendations based on caloric requirements [26]. A recent study used participant age, sex, and self-reported physical activity level to estimate caloric requirements and then used dietary recall data from 2003-2004 NHANES to estimate the percent of adolescents meeting current DGA recommendations for fruit and vegetable intake based on caloric requirements [17]. According to the study, 6.2% of adolescents aged 12-18 years met DGA fruit consumption recommendations, and 2.2% met vegetable consumption recommendations [17]. Only 0.9% met the DGA recommendations for both fruit and vegetable intake [17]. Dietary recommendations from other health organizations such as ACS and AICR/WCRF recommend five or more servings of fruits and vegetables daily [1,3]. In 2009, 22.3% of U.S. high school students nationwide had eaten fruit and vegetables five or more times per day during the seven days before the national YRBS survey [13]. The percentage of students who ate fruits and vegetables five or more times per day decreased significantly during 1999--2005 (23.9% - 20.1%; p < .05) and did not change significantly during 2005--2009 (20.1% - 22.3%) [13].

### Whole grains

Consumption of unprocessed whole grains is thought to be associated with a decreased cancer risk, and the 2005 DGA recommend consuming 3 or more 1-ounce-equivalent servings of grain daily, of which at least half should be whole grains [1,3,5,26]. Major sources of whole grains consumed among youth include yeast breads, popcorn, and ready-to-eat breakfast cereals [30]. NHANES data from 1999 to 2002 indicated that only 3.4% of adolescents 12-18 years met the DGA recommendations for whole grain consumption [18]. The national YRBS does not include questions about grain consumption [13].

### Healthy weight

Maintaining a healthy weight is also recommended for overall health and in support of cancer prevention [1-3,26-28]. When assessing the weight of U.S. children and adolescents, the 2000 CDC growth charts are often used [31]. The 2000 CDC growth charts are based on data from five cross-sectional, nationally representative health examination studies conducted during 1963-65, 1966-70, 1971-74, 1976-80, and 1988-94 [32]. The 2000 CDC growth charts provide height-for-age, weight-for-age, and BMI-for-age percentiles in girls and boys [32]. Although the definitions of childhood overweight and obesity vary somewhat in the current literature, experts recently recommended that children with a BMI greater than or equal to the 85^th ^percentile on the 2000 CDC growth charts of BMI-for-age (based on measured height and weight) be classified as overweight and children with a BMI greater than or equal to the 95^th ^percentile be classified as obese [33]. The prevalence of obesity among U.S. adolescents ages 12-19 was approximately 5.0% in the early 1970's, but prevalence rates reached 18.1% among adolescents during the 2007-2008 NHANES [34]. This increase in obesity over time has been observed across gender, racial, and ethnic groups [34]. Data from the 2009 national YRBS indicated that 12.0% of high school students nationwide were obese and 15.8% of students were overweight based on self-reported height and weight [13]. Significant linear increases occurred in the percentage of students who were obese (10.7% - 12.0%) and who were overweight (14.4% - 15.8%) during 1999 - 2009 [13].

### Energy-dense foods

Current AICR dietary guidelines for cancer prevention specifically recommend limiting energy-dense foods [1]. A diet rich in fruits, vegetables and whole grains, with limited amounts of refined sugars is likely to be low in energy-dense foods and conducive to maintaining a healthy weight. However, as can be seen from the statistics above, American youth are not consuming the recommended amounts of fruits, vegetables and whole grains, suggesting that they may be over-consuming more energy-dense foods [16,18,19].

### Refined sugars

Desserts, dairy desserts, and candy are substantial contributors of refined sugar to the diets of Americans [16,18,35]. Sugar-sweetened beverages (SSBs), including soda, sports drinks, fruit drinks and punches, low calorie drinks, sweetened tea, and other sweetened beverages, are another notable source of refined sugars in the diet. According to NHANES data from 1988 through 2004, approximately 80% of adolescents aged 12 - 19 years had consumed SSBs on the survey day [19]. Among those who consumed SSBs on the survey day, the mean amount consumed on the day of the survey increased from 30.3 ounces in 1988-1994 to 32.7 ounces in 1999-2004 [19]. The increase in SSB consumption appears primarily to have occurred among young males [19,36,37]. The national YRBS asks specifically about soda or pop consumption rather than all SSBs [13]. In the 2009 national YRBS, 29.2% of high school students nationwide had drunk a can, bottle, or glass of soda or pop [not including diet soda or diet pop] at least one time per day during the seven days before the survey [13]. Data from national YRBS indicates that this percentage has decreased in recent years, from 33.8% in 2007 to 29.2% in 2009 [13].

### Red and processed meat

Several recommendations encourage limiting consumption of red and processed meats [1-3,6]. The USDA estimates that, in general, Americans consume about 110 pounds (49.9 kilograms) of red meat per year, which equals to approximately 3 ounces per day [38,39]. Furthermore, data from both NHANES III (1988-1994) and NHANES 1999-2002 indicate that adolescents ages 12-16 consumed an average of 1-2 servings of red meat per day and just under one serving of processed meat per day [16]. The national YRBS does not include questions about meat consumption [13].

### Salt

Although dietary guidelines recommend limiting salt consumption, sodium intake among youth has increased over the past decade based on NHANES data [1,2,5,6,20-22,40]. This increase is likely due, in part, to the rise in the consumption of heavily processed foods, which tend to have high sodium contents [40]. The 2005 DGA recommend limiting sodium consumption to less than 2300 mg/day [18]. However, 2005-2006 NHANES data indicate males and females ages 12 - 19 years consumed approximately 4266 mg/day and 2950 mg/day respectively [20,25]. Youth were consuming slightly less sodium in 2001-2002 according to NHANES data (3990 mg/day among males and 2831 mg/day among females) but were still above the DGA recommendation [22]. Salt consumption is not assessed on the national YRBS [13].

### Alcohol

Dietary recommendations also address alcohol consumption and generally state that if alcoholic beverages are consumed, it should be done in moderation [1-3,5]. However, the 2005 DGA state alcohol should not be consumed by children and adolescents [26]. Furthermore, an early age at first drink (specifically under the age of 18) may be associated with an increased risk of alcohol dependence and other alcohol use disorders in adulthood [41]. In 1984, the Federal Uniform Drinking Age Act (Public Law 98-363) required states to enforce an age minimum of 21 years for purchasing and/or publically possessing alcoholic beverages. Although some literature suggests that this law was associated with a decrease in alcohol consumption among minors [42], youth in the U.S. continue to have access to alcohol [13,43]. NHANES data from 1999-2004 showed 39% of adolescents aged 12-17 years had at least one drink of alcohol in their lifetime, and 21% had at least one drink of alcohol during the 30 days before the survey [43]. According to data from the 2009 national YRBS, 72.5% of high school students nationwide had had at least one drink of alcohol on at least one day during their life, and 41.8% of students had at least one drink of alcohol on at least one day during the thirty days before the survey (i.e., current alcohol use) [13]. During 1999 - 2009, the percentage of students who reported current alcohol use decreased (50.0%-41.8%) [13].

### Mycotoxins

Cancer prevention guidelines also recommend avoiding consumption of moldy grains and legumes which may be contaminated with mycotoxins, particularly aflatoxin [1,2,6]. Aflatoxin is one of many naturally occurring mycotoxins which can grow on agricultural commodities such as grains and nuts in the field and in storage and is considered to be a known carcinogen [1,2,6,43]. Ochratoxin and fumonisin are other mycotoxins classified by IARC as "possibly carcinogenic" to humans [44]. The FDA has established action levels to control the levels of mycotoxins in food and animal feed, but there is no system in place to monitor exposure among adolescents in the United States [45].

### Arsenic

AICR recommends avoiding the use of any source of water that may be contaminated with arsenic (Table 2). Arsenic is classified by IARC as "carcinogenic to humans," and exposure to arsenic within the general U.S. population can occur not only through water consumption but also through consumption of contaminated meats, grains, produce and seafood [24,44]. According to the *Fourth National Report on Human Exposure to Environmental Chemicals*, the mean urinary arsenic levels [in µg/L] among children ages 6-11 years and 12 - 19 years were 7.08 (5.66-8.84) and 8.55 (7.34-9.97) respectively [24].

## Conclusion

Dietary guidelines for cancer prevention are similar across prominent public health organizations. In spite of a general scientific consensus on the importance of a healthy diet for cancer prevention, the actual diets of a large proportion of America's youth are not aligned with these dietary recommendations. Pre-adolescents and adolescents tend to fall short of recommendations for fruit, vegetable, and whole grain consumption. Furthermore, an unhealthy proportion of their diet is composed of energy-dense foods that are high in fat (such as red and processed meats), refined sugar (such as refined grains and SSBs), and/or salt.

Improving dietary behaviors poses many challenges. Among youth, dietary behaviors are influenced by numerous factors at multiple levels: individual, interpersonal, community, and society levels [11]. Identifying key influential factors may inform future public health policies and practices. For example, adolescents generally do not have the same level of control over their food intake that adults do, and much of the responsibility to support healthy eating habits falls on parents [46]. Parents tend to be influenced by socioeconomic factors, and changes to food pricing offer a potential strategy for dietary improvements [11]. Another example is the school food environment which plays a key role in proper nutrition. Millions of children and adolescents rely on federally-supported school breakfast and lunch programs [47,48]. Yet participation in the school meal program has been shown to be associated with an increased prevalence of excessive sodium intake [49]. Such findings suggest that improvements to the school meal program alone could have a positive impact on the quality of youth's diets.

When assessing the dietary behaviors among youth related to cancer prevention, we face several limitations. First, dietary intake data from both NHANES and YRBS rely heavily on self-reported information, which can be subject to bias and error. NHANES dietary intake data are collected via multiple 24-hour dietary recalls which may be less prone to error than other dietary recall methods [50]. NHANES data for BMI is based on height and weight measurements which are considered more accurate than self-reported height and weight, as are used in the national YRBS [51]. Second, dietary recommendations for cancer prevention tend to be based on knowledge related to adults' dietary behaviors, and there is insufficient evidence to know how strongly dietary habits during childhood predict lifetime dietary patterns [52-55]. Epidemiological studies suggest that unhealthy dietary habits that develop early in life and persist into adulthood may increase the risk of some cancer types, but additional research is needed to better understand the degree to which childhood dietary patterns relate to subsequent cancer risk later in life [56]. Additionally, the relationship between cancer and certain diet-related factors may not necessarily be the same throughout the lifespan, as has been observed for obesity and breast cancer [57,58]. Third, our understanding of the risks associated with potential carcinogens in the diet are often based on the effects of such contaminants in adults. These contaminants may be metabolized differently in a child's or adolescent's body due to differences in body size and composition, and youth have a "unique vulnerability" to the effects of chemical, physical, and biological agents [59-61]. This difference may have implications for age-specific recommendations regarding potential carcinogens in youth diets.

Cancer etiology and cancer risk assessment are dynamic fields of scientific investigation, and our understanding about the role of diet in cancer occurrence is continually evolving. As a result, the dietary recommendations for cancer prevention examined in this review may change over time. For example, although the consumption of fruits and vegetables has long been associated with lower risks for certain types of cancers, recent research suggests that the consumption of fruits and vegetables may not be as protective against cancer as had been previously assumed [62,63]. The potential cancer risks associated with low levels of exposure to certain chemicals are being reassessed in the face of evidence of upstream effects such as changes in hormone levels and immune function [64]. Such chemicals are not addressed in the major dietary guidelines for cancer prevention, and more research is needed to determine if the levels of exposure to these contaminants in the United States is cause for a health concern [24]. Research evaluating the health effects of these chemicals is particularly important to protect the health of children, who are generally thought to be more susceptible to carcinogens than adults [65]. In the future, further advances in our scientific knowledge may reduce current uncertainties about diet and cancer and present new opportunities to reduce cancer risk.

This review illustrates discrepancies between expert recommendations about diet and cancer and actual dietary practices among youth and points to the need for more research to better promote the translation of science into practice. Data from the 2003 Health Information National Trends Survey (HINTS) suggests that at least half of the general population in the U.S. is aware that a healthy diet may reduce one's cancer risk [66]. Although education and awareness about healthy eating for cancer prevention are important, interventions that make healthy options the default choice regardless of education, income, or other societal factors, change the environmental context in which dietary choices are made and may have a greater impact on the eating behaviors of America's youth [67]. Future research should focus on developing and evaluating policies and interventions at the community, state and national levels for aligning the diets of American youth with the evolving scientific evidence regarding the role of diet in cancer incidence, as well as monitoring the long-term impact of different dietary patterns in childhood on cancer incidence later in life. If such public health efforts were successful, it could potentially reduce future cancer rates and improve the overall health of America's youth [4,11].

## Competing interests

The authors declare that they have no competing interests.

## Authors' contributions

DH participated in the conception and design of the paper, carried out the initial literature review, and contributed to drafting and critically revising the manuscript. MW participated in the conception and design of the paper and contributed to drafting and critically revising the manuscript. Both authors have read and approved the final manuscript.
